# Comparison of short-term outcomes between laparoscopically-assisted vs. transverse-incision open right hemicolectomy for right-sided colon cancer: a retrospective study

**DOI:** 10.1186/1477-7819-5-49

**Published:** 2007-05-11

**Authors:** Varut Lohsiriwat, Darin Lohsiriwat, Vitoon Chinswangwatanakul, Thawatchai Akaraviputh, Narong Lert-akyamanee

**Affiliations:** 1 Department of Surgery, Faculty of Medicine Siriraj Hospital, Mahidol University, Bangkok 10700, Thailand

## Abstract

**Background:**

Laparoscopically-assisted right hemicolectomy (LRH) is an acceptable alternative to open surgery for right-sided colon cancer which offers patients less pain and faster recovery. However, special equipment and substantial surgical experience are required. The aim of the study is to compare the short-term surgical outcomes of LRH and open right hemicolectomy through right transverse skin crease incision (ORHT) for right-sided colon cancer.

**Patients and methods:**

This retrospective study included 33 patients with right-sided colon cancer who underwent elective right hemicolectomy by laparoscopic or open approaches through right transverse skin crease incision between March 2004 and September 2006 at the Department of Surgery, Faculty of Medicine Siriraj Hospital. Operative details, postoperative requirement of narcotics, recovery of bowel function, and oncological parameters were analyzed.

**Results:**

Thirteen patients underwent LRH and 20 patients underwent ORHT. Both approaches achieved adequate oncological resection of the tumor. The laparoscopic group were characterized by shorter average incision lengths (7.7 vs 10.3 cm; p < 0.001), but longer average operating times (208 vs 105 min; p < 0.001). There was no significant difference in the time to first bowel movement, time to defecation, and time to resumption of normal diet between both groups (59 vs 64 hr; p = 0.64, 3.2 vs 3.7 d; p = 0.25 and 3.9 vs 4.3 d; p = 0.39). There was no statistically significant difference in the time to discontinuation of intravenous nacrotics and the length of hospital stay (1.0 vs 1.4 d; p = 0.25 and 6.2 vs 7.1 d; p = 0.3).

**Conclusion:**

LRH and ORHT for right-sided colon cancer resulted in the same short-term surgical outcomes including postoperative bowel function, narcotics consumption and length of hospital stay. However, LRH required a significantly longer operating time.

## Background

Colorectal cancer is one of the leading causes of cancer death in Thailand. Right-sided colon cancer represents one third of all cases and is conventionally treated by right hemicolectomy. Most surgeons prefer to perform the operation via midline incision. Laparoscopically-assisted right hemicolectomy (LRH) is an effective operation which decreases postoperative pain and shortens hospital stay [1-3]. However, LRH is quite a complicated operation requiring highly skilled surgeons as well as specialized instrumentation.

A recent publication has suggested that exploratory laparotomy via transverse skin crease incision can provide benefits in terms of ease of operation, reduced postoperative pain, earlier return of bowel function and more rapid discharge from hospital [4]. However, no comparison of the surgical outcomes of these alternative surgical approaches has so far been reported.

The aim of this study is to compare the short-term surgical outcomes of laparoscopically-assisted right hemicolectomy (LRH) and open right hemicolectomy through right transverse skin crease incision (ORHT) for right-sided colon cancer.

## Patients and methods

After obtaining approval from the institutional ethics committee, we carried out a retrospective analysis of patients with right-sided colonic adenocarcinoma who underwent LRH or ORHT between March 2004 and September 2006 at the Department of Surgery, Faculty of Medicine Siriraj Hospital, Bangkok, Thailand. Only American Society of Anesthesiologists (ASA) class I and II patients undergoing elective curative procedures were included. Curative procedures were defined as those in which there was no pre- or intra- operative evidence of distant metastasis and there was no postoperative macroscopic residual tumor.

Patients who were immunocompromised, receiving antiplatelet or anticoagulant drugs, or who had a history of previous intra-abdominal operations, recurrent tumors, adjacent organ resection, or acute complicated conditions such as colonic obstruction or perforation, perioperative epidural analgesia or failed laparoscopic procedure that needed conversion to open surgery were excluded.

Patients were interviewed to establish their medical history and a thorough physical examination was performed. Preoperative investigation included barium enema, complete colonoscopy with biopsy, chest X-ray, ultrasonography or CT scan of upper abdomen, and relevant serum tests.

All operations were performed by one of the authors. Type of the operation was depended on the surgeon's preference. A signed informed consent was provided by every patient. Each patient underwent preoperative mechanical bowel preparation using 2 liters of polyethylene glycol a day before surgery. All patients received general anesthesia. Intravenous prophylaxis antibiotics were also administrated.

In the cases receiving LRH, a vertical midline 1.5 cm incision was made just above the umbilicus for zero-degree camera port, and another three 0.5 cm incisions were made for instrumental trocars. The camera port was extended upward for extracorporeal ileocolonic anastomosis at the end of the operation. In the cases receiving ORHT, an incision was made along the skin crease on the right side of abdomen, about 1 cm above the umbilicus.

A standard oncological right hemicolectomy with high vessel ligation, wide excision and stapled side-to-side ileocolonic anastomosis was performed on all patients in both groups. The incision was closed in layers. No intra-abdominal drain or nasogastric tube was used. Prophylactic intravenous antibiotics were discontinued within 24 hours.

Routine postoperative care was provided for each patient. The time elapsing before first bowel movement (passing flatus) was recorded by nursing staff. Patients were allowed oral fluids if passing flatus. Resumption of normal diet was decided by agreement between surgeons and patients. Patients were discharged from the hospital when they displayed no fever, good appetite and satisfactory mobility. All patients were scheduled for postoperative follow-up 30 days later.

The data recorded included patients' demographic and operative details (length of incision, operating time, blood loss and postoperative complications), recovery details (time to first bowel movement, time to defecation, time to resumption of normal diet, time to discontinuation of intravenous narcotics and length of hospital stay) and oncological details (tumor size, lymph node harvest, resection margin).

Data were complied using a SPSS computer program (version 10.0 for Windows) and the Kolmogorov-Samirnov test was used to evaluate the data distribution. An unpaired t-test was used to compare data between the two groups of patients when these were found to be in normal distribution pattern. The Mann-Whitney U test was used when this was not the case. A p-value of less than 0.05 was considered statistically significant.

## Results

Thirty-six patients were enrolled. After the application of exclusion criteria (1 patient with liver metastasis, 2 patients receiving perioperative epidural analgesia), 33 patients were left for the study. Thirteen patients underwent LRH and 20 patients underwent ORHT.

An analysis of the demographic data, operative details and oncological parameters revealed no statistically significant difference between two groups, except length of incision and operating time (Table 1).

**Table 1:** Demographic data, operative details and oncological parameters (mean ± SD).

	**LRH (n = 13)**	**ORHT (n = 20)**	**p value**
**Age (years)**	56.9 ± 13.5	65.2 ± 16.0	0.13
**Female (%)**	53.8	65.0	0.78
**Body Mass Index (kg/m2)**	20.8 ± 1.8	20.7 ± 4.2	0.93
**Serum albumin (g/dl)**	3.5 ± 0.4	3.3 ± 0.5	0.33
**Length of incision (cm)**	7.7 ± 1.1	10.3 ± 2.0	<0.001
**Operating time (min)**	207.7 ± 56.7	104.5 ± 24.2	<0.001
**Estimated blood loss (ml)**	120.8 ± 57.9	107.5 ± 40.6	0.48
**Tumor size (cm)**	5.7 ± 2.7	6.1 ± 2.6	0.74
**Nodes harvested (n)**	29.2 ± 18.1 (range 5–66)	18.8 ± 10.8 (range 7–47)	0.08
**TMN Stage (n and %)**			0.76
**1**	1 (8%)	2 (10%)	
**2**	2 (15%)	7 (35%)	
**3**	10 (77%)	11 (55%)	
**Positive resection margins (n)**	0	0	

No re-operation or re-admission occurred within 30 days for any patient. No thirty-day postoperative mortality was reported. One case of superficial surgical site infection was reported in the ORHT group. Both approaches achieved adequate oncological resection of the tumor. The LRH group were characterized by shorter incisions (7.7 vs. 10.3 cm; p < 0.001), but longer operating times (208 vs. 105 min; p < 0.001). There were no significant differences in: time to first bowel movement (59 vs. 64 hr; p = 0.64); time to defecation (3.2 vs. 3.7 d; p = 0.25); time to resumption of normal diet (3.9 vs. 4.3 d; p = 0.39); time to discontinuation of intravenous narcotics (1.0 vs. 1.4 d; p = 0.25); and length of hospital stay (6.2 vs. 7.1 d; p = 0.3) (Table 2).

**Table 2:** Postoperative complications and recovery parameters (mean ± 2 SD).

	**LRH (n = 13)**	**ORHT (n = 20)**	**p value**
**Postoperative complication (n)**	0	1	1.0
**Time to first bowel movement (hours)**	59.1 ± 23.2	63.6 ± 28.4	0.64
**Time to defecation (days)**	3.2 ± 0.9	3.7 ± 1.8	0.25
**Time to resumption of normal diet (days)**	3.9 ± 1.0	4.3 ± 1.1	0.39
**Time to discontinuation of intravenous narcotics (days)**	1.0 ± 0.9	1.4 ± 1.0	0.25
**Length of hospital stay (days)**	6.2 ± 2.4	7.1 ± 2.6	0.30

## Discussion

The main benefits of laparoscopic gastrointestinal surgery are reduction of postoperative pain and shortened hospital stay. LRH can be performed through multiple small incisions with the assistance of advanced laparoscopic equipment. However, the learning curve of this complex procedure is demanding [5]. It is therefore recommended that this procedure should be performed only by experienced surgeons in order to ensure complete oncological resection [6].

Several articles have claimed that gastrointestinal cancer surgery, including colectomy, can be performed through one small incision, known as minilaparotomy, with the same results as conventional laparotomy. Hsu [7] has reported performing a wide range of colectomies through a skin incision of less than 7 cm in 316 patients with various colorectal diseases. He observes that small incisions do not prolong the operating time. Nakagoe *et al*., [8] has reported 86% success rate for colectomy through minilaparotomy for colon cancer. The two most common reasons for failure were tumor adhesion or invasion into adjacent organs and inability to divide the lienocolic and phrenocolic ligaments at the splenic flexure. In general, this minilaparotomy approach seems to be easily performed because surgeons are familiar with open surgery and no complex instruments are required.

Laparotomy can be done either through midline or transverse incision. There is some evidence that a transverse incision may be accompanied by less postoperative pain and less impact on pulmonary function [9,10], while offering adequate exposure of the operative field. Right transverse skin crease incision is a favored approach for right hemicolectomy because surgeons can easily take down hepatic flexure and fully mobilize the right-sided colon. The ileocolonic anastomosis can be done through this incision without any tension.

In the present study, we found no significant difference in recovery of postoperative bowel function, narcotics requirement, and length of hospital stay between LRH and ORHT for right-sided colon cancer. The less invasive nature of both these surgical techniques is clearly desirable. The mean length of incision in ORHT is comparable to that of hand-assisted laparoscopic colectomy in other studies [11,12]. It is widely recognized that such small incision are associated with reduced systemic inflammatory response [13].

Some researchers have argued that the recovery of postoperative bowel function occurs earlier following laparoscopically-assisted colectomy than open colectomy [14,15]. However, other investigations [16-18] and our study reveal no such benefit. Perhaps this is because recovery of bowel function is more associated with the quantity of narcotics prescribed rather than the type or length of the incision used [19]. Time to discharge is also influenced by many factors, such as the patient's socioeconomic status and their perception of their own postoperative recovery. In the COST study [6], the median length of hospital stay in the laparoscopic group was 5 days which was only one day shorter than the open group. Kuhry *et al*., [20] has also demonstrated that the size of the case load borne by the hospital has a significant impact on short-term outcomes for these operations.

The present study has demonstrated that both surgical techniques can adequately remove mesenteric lymph nodes and achieve a good margin of resection. LRH seemed to be able to harvest greater numbers of lymph nodes. This can probably be explained by the better videoscopic access it provides during deep dissection. However, laparoscopically-assisted colectomy significantly increased operating time, confirming the uniform testimony of other major studies [15,21,22]. It is thus possible that this increase in operating time for LHR might offset its benefits. However, a rather small sample was assessed in this study and the surgeons involved were comparatively inexperienced in laparoscopically-assisted colectomy. A prospective randomized comparison of the ORHT and LRH performed by highly skilled laparoscopic surgeons is really needed before a definite conclusion can be drawn on this point. What our findings do highlight is that ORHT may be considered as a viable alternative approach offering minimally invasive surgery if surgeons are unfamiliar with LRH.

## Conclusion

The results of the study demonstrated that LRH and ORHT for right-sided colon cancer resulted in the same short-term surgical outcomes including postoperative bowel function, narcotics consumption and length of hospital stay, without compromising the standards of tumor resection. However, LRH had a significantly longer operative time.

## Competing interests

The author(s) declare that they have no competing interests.

## Authors' contributions

**VL **is the principle investigator who contributed to acquisition of data and analysis as well as performed both laparoscopic and open surgery. **DL **conceived the study and performed open surgery. **VC **carried out laparoscopic surgery and critically revised the manuscript. **TA **participated in the design of the study and performed open surgery. **NL **drafted the manuscript and carried out laparoscopic surgery. All authors read and approved the final manuscript.
